# Exploring Vaccine Hesitancy Among Parents in a Rural Pediatric Clinic System: A Statistical Study

**DOI:** 10.7759/cureus.64864

**Published:** 2024-07-18

**Authors:** Masab A Mansoor, David J Grindem, Nicholas Kidd

**Affiliations:** 1 Pediatrics, Edward Via College of Osteopathic Medicine, Alexandria, USA; 2 Family Medicine, Mayo Clinic, Rochester, USA; 3 Family Medicine, University of Virginia, Charlottesville, USA

**Keywords:** rural pediatric population, vaccine hesitancy and belief, rural pediatrics, pediatric vaccines, vaccine hesitancy

## Abstract

Background: Vaccine hesitancy has been a growing concern in the United States, particularly in rural areas where access to healthcare services may be limited. A rural pediatric clinic system in central Louisiana serves a population with historically low childhood immunization rates. This study explored the prevalence and determinants of vaccine hesitancy among parents of pediatric patients at the organization's clinics.

Methods: A qualitative survey was conducted among parents who declined vaccines for their children at the organization's clinics. The survey collected information on parents' attitudes, beliefs, decision-making processes regarding childhood vaccinations, and demographic information about the parents, including income and education levels. Thematic analysis was used to identify key themes and patterns in the survey responses.

Results: Thirty out of 47 parents (response rate: 64%) completed the survey. Most respondents (n=24, 80%) expressed concerns about vaccine safety and potential side effects. Many parents (n=16, 60%) cited information from social media and alternative health sources as influencing their decision to decline vaccines. Religious and philosophical beliefs were also common reasons for vaccine refusal (n=13, 43%). Another significant theme was the lack of trust in healthcare providers and the pharmaceutical industry (n=17, 53%). No significant differences in responses were observed based on the parent's race or the child's sex. Ninety percent of participants (n=27) reported a household income of under $50,000, and 87% of participants (n=26) had a high school education or less.

Conclusion: Vaccine hesitancy among parents in this rural pediatric population appears to be driven by concerns about vaccine safety, exposure to misinformation, religious and philosophical beliefs, and distrust in the healthcare system. Addressing these factors through targeted education, provider communication, and community engagement may be essential for improving childhood immunization rates in this vulnerable population. The findings highlight the need for culturally sensitive, evidence-based interventions to combat vaccine hesitancy in rural communities.

## Introduction

Vaccine hesitancy, defined as the reluctance or refusal to vaccinate despite the availability of vaccines, has been a growing concern worldwide [1]. The World Health Organization has identified vaccine hesitancy as one of the top ten global health threats [2]. Vaccine hesitancy can lead to decreased immunization rates, an increased risk of vaccine-preventable disease outbreaks, and significant public health consequences [3].

In the United States, childhood immunization rates have been declining in recent years, with a growing number of parents expressing concerns about vaccine safety and necessity [4]. This trend is particularly pronounced in rural areas, where access to healthcare services may be limited and exposure to vaccine misinformation may be higher [5]. Rural populations often face unique challenges contributing to vaccine hesitancy, such as geographic isolation, lower socioeconomic status, and cultural beliefs [6].

A rural pediatric clinic system in central Louisiana serves a particularly vulnerable population to the consequences of vaccine hesitancy. The region has historically faced challenges in achieving optimal childhood immunization rates, with some parishes reporting rates well below the state average [6]. Understanding the factors contributing to vaccine hesitancy in this population is crucial for developing targeted interventions to improve immunization rates and protect children's health in the community.

This study aims to investigate the prevalence and determinants of vaccine hesitancy among parents of pediatric patients at a rural health pediatric clinic in central Louisiana. By examining the socioeconomic, cultural, and attitudinal factors associated with vaccine hesitancy in this rural population, we seek to inform the development of evidence-based strategies to address this critical public health issue. The findings of this study may have implications for other rural pediatric practices facing similar challenges and contribute to the growing body of literature on vaccine hesitancy in underserved communities.

## Materials and methods

Study design and setting

This qualitative study was conducted over three months, from August 2023 to October 2023, at a rural pediatric clinic system with four locations in central Louisiana. The study employed a cross-sectional survey design to explore vaccine hesitancy among parents who declined vaccines for their children during clinic visits. 

Participants and recruitment

Participants were eligible for the study if they were parents or legal guardians of patients aged 0-18 who had declined one or more recommended childhood vaccines while visiting one of the organization's clinics. Purposive sampling was used to recruit participants, with clinic staff identifying eligible parents during routine visits and inviting them to participate in the study. Recruitment continued until data saturation was reached.

Sample Size Justification

In qualitative research, the goal is to reach data saturation, the point at which no new themes or information emerge from additional interviews [7]. While there is no universally accepted sample size for qualitative studies, researchers have provided guidance based on the study design and objectives. For this study, we aimed to recruit a minimum of 20 participants, which has been suggested as a suitable sample size for achieving data saturation in qualitative research involving semi-structured interviews [8]. We also considered factors such as the population's homogeneity, the research question's complexity, and the available resources in determining our target sample size [9]. Recruitment continued until data saturation was reached after 30 participants had completed the survey.

Data collection

Data were collected using a qualitative survey consisting of open-ended questions. The research team developed the survey based on a review of the literature on vaccine hesitancy and input from pediatric healthcare providers. The survey questions explored parents' attitudes, beliefs, decision-making processes regarding childhood vaccinations, and their sources of information and trust in the healthcare system. The survey was administered online using the Qualtrics platform. Eligible parents were provided with a link to the survey and could complete it at their convenience using a computer or mobile device. The survey took approximately 15-20 minutes to complete.

To ensure the validity of the survey instrument, the questionnaire was developed based on a comprehensive review of the literature on vaccine hesitancy and was reviewed by a panel of experts in the field. The expert panel consisted of healthcare professionals with experience in vaccine hesitancy, pediatric care, and survey design. The panel provided feedback on the clarity, relevance, and comprehensiveness of the survey questions, and their suggestions were incorporated into the final version of the questionnaire. Additionally, a pilot test of the survey was conducted with a small sample of five parents to assess the clarity and comprehensibility of the questions and to estimate the time required to complete the survey.

Data analysis

Survey responses were analyzed using thematic analysis, a qualitative data analysis method that involves identifying, analyzing, and reporting patterns or themes within the data [10]. The analysis was conducted iteratively, with the researchers moving back and forth between the phases to refine the themes and ensure a comprehensive understanding of the data.

The first phase of thematic analysis involved familiarization with the data. The researchers read through the survey responses multiple times to immerse themselves in the data and gain a broad understanding of the participants' perspectives and experiences. The researchers generated initial codes in the second phase by systematically identifying and labeling meaningful data segments. They conducted the coding process independently to ensure reliability and reduce potential bias. The third phase involved searching for themes by collating the initial codes into potential themes and gathering all relevant data for each theme. The researchers examined the relationships between codes and themes to develop a coherent and meaningful structure. In the fourth phase, the researchers reviewed and refined the themes. This phase involved checking the themes against the coded extracts and the entire dataset to ensure they accurately represented the data. Themes were merged, split, or discarded as necessary to create a clear and comprehensive thematic map. The researchers defined and named the themes in the fifth phase. This phase involved identifying each theme's essence and determining what aspect of the data it captured. The researchers also developed clear and concise names for each theme that effectively communicated its content and meaning. The final phase involved producing the report, which included selecting compelling extract examples, relating the analysis to the research question and literature, and producing a scholarly report of the findings.

Throughout the analysis process, the researchers engaged in ongoing discussion and reflection to challenge assumptions, refine interpretations, and ensure the trustworthiness of the findings. NVivo 12 qualitative data analysis software was used to organize and manage the data, facilitating the iterative coding and theme development processes.

Ethical considerations

The study was approved by the ethics committee of the pediatric practice (approval number: 14291). Informed consent was obtained from all participants prior to their completion of the survey. Participants were informed that their participation was voluntary and that they could withdraw from the study at any time. All data were kept confidential and stored on secure servers.

Avoiding Bias

Throughout the analysis process, several measures were taken to avoid bias and ensure the trustworthiness of the findings. Using a structured codebook and the involvement of two independent coders helped maintain consistency and reduce the potential for individual biases in the interpretation of the data [11]. Regular meetings between the coders were held to discuss and resolve any discrepancies in the coding, ensuring a high level of inter-coder agreement [12].

To further enhance the credibility of the findings, member checking was conducted by sharing a summary of the themes with a subset of participants and soliciting their feedback [13]. This process allowed for verifying the researchers' interpretations and ensured that the findings accurately reflected the participants' experiences and perspectives. Additionally, using reflexivity throughout the research process helped minimize the influence of researcher bias [14]. The researchers regularly discussed and documented their backgrounds, assumptions, and potential biases and actively sought alternative explanations and negative cases during the analysis to challenge their initial interpretations. By employing these strategies, the researchers aimed to maintain the integrity and trustworthiness of the qualitative data analysis, reduce the potential for bias, and ensure that the findings accurately represented the participants' views and experiences.

## Results

Participant characteristics

Table 1 presents the participant characteristics (n=30), including the response rate, relationship to the child, age, race/ethnicity, number and age of children, annual household income, and education level.

**Table 1:** Participant characteristics (N=30)

Characteristic		N
Relationship to child	Mother	27 (90%)
	Father	3 (10%)
Race/ethnicity	White	23 (77%)
	Black or African American	6 (20%)
	Other	1 (3%)
Annual household income	Less than $35,000	7 (23%)
	$35,000–50,000	20 (67%)
	More than $50,000	3 (10%)
Education level	Less than high school	6 (20%)
	High school diploma or equivalent	20 (67%)
	Some college or vocational training	2 (7%)
	Bachelor’s degree or higher	2 (7%)
Number of children, median (range)		2 (1–5)
Age of children, mean (range)		6 (1–6)
Age, mean (range)		32 (22–45)

Thematic analysis

The thematic analysis of survey responses revealed four main themes related to vaccine hesitancy among parents in this rural pediatric population: (i) concerns about vaccine safety and side effects; (ii) the influence of social media and alternative health sources; (iii) religious and philosophical beliefs; and (iv) distrust in the healthcare system. Table 2 provides the prevalence of these themes, and Table 3 describes the four main themes related to vaccine hesitancy among the study participants and their perspectives.

**Table 2:** Prevalence of themes related to vaccine hesitancy among parents in a rural pediatric clinic system (n=30)

Theme	n (%)
Concerns about vaccine safety and side effects	24 (80%)
Influence of social media and alternative health sources	18 (60%)
Religious and philosophical beliefs	13 (43%)
Distrust in the healthcare system	17 (56%)

**Table 3:** Common themes related to vaccine hesitancy among parents in a rural pediatric clinic and participant perspectives

Theme	Description	Participant Perspectives
Concerns about vaccine safety and side effects	Parents expressed worries about potential adverse reactions, long-term health consequences, and the perceived risk-benefit ratio of vaccines.	"I've heard so many stories about kids getting autism after getting their shots. I just can't take that risk with my child. I worry about the long-term effects of putting all those chemicals into my baby's body."
Influence of social media and alternative health sources	Parents cited information from Facebook groups, parenting forums, natural health websites, and other online sources as shaping their vaccine hesitancy.	"I've done a lot of research online and found a lot of evidence that vaccines are harmful. I trust the opinions of other parents who have been through this more than I trust the doctors. I follow a lot of natural health pages on Facebook, and they've really opened my eyes to the risks of vaccines."
Religious and philosophical beliefs	Some parents held religious or personal beliefs that conflicted with vaccination, such as the idea that vaccines are unnatural or that God intended for children to develop immunity through natural exposure.	"I believe that God created our bodies to heal themselves naturally. Vaccines go against that belief. I don't think it's right to put foreign substances into our children's bodies. We should trust in nature and our own immune systems."
Distrust in the healthcare system	Parents expressed skepticism about the motives of healthcare providers and pharmaceutical companies, believing that profit and policy, rather than patient well-being, drive vaccine recommendations.	"I don't trust the doctors to give me all the information. They just want to push vaccines because that's what they're told to do. I think there's a lot of corruption in the pharmaceutical industry. They're more interested in making money than keeping our kids safe."

The two tables provide an overview of the main themes from the survey responses and their prevalence among the study participants. Table 2 shows that the four most common themes related to vaccine hesitancy in this rural pediatric population were concerns about vaccine safety and side effects (n=24, 80%), the influence of social media and alternative health sources (n=18, 60%), religious and philosophical beliefs (n=13, 43%), and distrust in the healthcare system (n=17, 56%).

Table 3 delves deeper into these themes, providing a detailed description of parents' concerns, beliefs, and experiences and representative quotes that illustrate these perspectives. The table highlights the emotional nature of parents' fears about vaccine safety, the role of online information sources in shaping vaccine attitudes, the impact of personal and religious beliefs on vaccine decision-making, and the underlying distrust in the motives of healthcare providers and pharmaceutical companies.

## Discussion

This qualitative study explored the prevalence and determinants of vaccine hesitancy among parents in a rural pediatric clinic system in central Louisiana. The findings suggest that vaccine hesitancy is a significant concern in this population, with many parents expressing doubts about the safety and necessity of childhood vaccinations. Tables 2-3 paint a complex picture of the factors contributing to vaccine hesitancy in this rural pediatric population. The high prevalence of safety concerns and the influence of alternative information sources underscore the need for practical, evidence-based communication strategies to address parents' fears and counteract misinformation. The role of religious and philosophical beliefs and distrust in the healthcare system also suggests that building trust and understanding between healthcare providers and parents is crucial for improving vaccine acceptance.

The themes identified in this study are consistent with previous research on vaccine hesitancy in rural populations. A study conducted in rural Oregon, USA, found that concerns about vaccine safety and potential side effects were a significant barrier to vaccine acceptance [15]. Similarly, a study in rural India highlighted the influence of social networks and alternative health beliefs on vaccine decision-making [16]. 

Religious and philosophical beliefs are also essential to vaccine hesitancy [10]. A study in rural Ohio, USA, found that parents who exempted their children from school immunization requirements often cited religious or philosophical objections [17]. A study of vaccine hesitancy in rural Alberta, Canada, reported that some parents believed that natural immunity was preferable to vaccination [18].

Distrust in the healthcare system and pharmaceutical industry has been identified as a significant predictor of vaccine hesitancy in various settings. A study of vaccine-hesitant parents in rural Colorado, USA, found that a lack of trust in healthcare providers and government agencies was a significant barrier to vaccine acceptance [19]. Similarly, a study in rural Scotland highlighted parents' concerns about the financial motivations of pharmaceutical companies and the adequacy of vaccine testing and regulation [20].

The findings of this study have important implications for public health practice and policy. Addressing vaccine hesitancy in rural populations will require a multifaceted approach, considering the unique social, cultural, and economic factors shaping attitudes toward vaccines in these communities. Healthcare providers play a critical role in promoting vaccine acceptance, and strategies to improve provider communication and build trust with vaccine-hesitant parents are essential [21-22]. Community-based interventions that engage local leaders, faith communities, and other trusted sources of information may also be effective in increasing vaccine uptake [23-24].

This study has several limitations that should be noted. The small sample size and single geographic location limit the generalizability of the findings to other rural populations. The use of a qualitative survey methodology may not have captured the full range of factors influencing vaccine hesitancy, and more in-depth interviews or focus groups may have yielded additional insights. While the study collected information on participants' income and education levels, the small sample size limited the ability to perform subgroup analyses based on these factors. Future research with larger, more diverse samples may provide valuable insights into the socioeconomic factors influencing vaccine hesitancy in rural populations. Finally, the study relied on self-reported data, possibly subject to social desirability bias.

Despite these limitations, this study contributes to the literature on vaccine hesitancy in rural populations. By identifying the key themes and patterns of vaccine hesitancy in this vulnerable population, the study highlights the need for targeted interventions to address the specific concerns and beliefs that drive vaccine refusal. Future research should explore the effectiveness of different strategies for promoting vaccine acceptance in rural communities, including provider education, community outreach, and social media campaigns. Ultimately, improving childhood immunization rates in rural areas will require a sustained and collaborative effort by healthcare providers, public health professionals, and community leaders to build trust, combat misinformation, and ensure equitable access to vaccines for all children.

## Conclusions

This study provides insights into the prevalence and determinants of vaccine hesitancy among parents in a rural pediatric clinic system in central Louisiana. The findings suggest that vaccine hesitancy is a complex issue driven by concerns about vaccine safety, exposure to misinformation, religious and philosophical beliefs, and distrust in the healthcare system. The high prevalence of vaccine hesitancy in this population is concerning, as it may lead to decreased immunization rates and an increased risk of vaccine-preventable diseases. Addressing vaccine hesitancy in rural populations requires a comprehensive, culturally sensitive approach that considers the unique factors shaping attitudes toward vaccines in these communities and involves healthcare providers, community leaders, and trusted information sources to promote vaccine acceptance. The findings have implications for public health practice and policy, highlighting the need for targeted interventions and future research to address the specific concerns and beliefs driving vaccine hesitancy in rural populations.

## Appendices

**Table 4:** Survey instructions and questions

Survey Instructions	
Dear Parent or Guardian, You are invited to participate in a research study exploring parents' attitudes, beliefs, and decision-making processes regarding childhood vaccinations. Your perspective is invaluable in helping us understand the factors that influence vaccine acceptance or hesitancy in our community. By completing this survey, you will contribute to our efforts to improve childhood immunization rates and protect the health of children in our region. The survey will take approximately 10-15 minutes to complete. Your participation is entirely voluntary, and you may choose to skip any questions that you do not feel comfortable answering. All responses will be kept strictly confidential and used only for research purposes. No identifying information will be collected or linked to your responses.	
Survey Questions	Answer Choices
Do you agree to participate in this study?	Agree/Disagree
What is your relationship to the child?	Mother, Father, Legal Guardian, Other
What is your race/ethnicity?	White, Black or African American, Hispanic or Latino, Asian, Native American, Other​​​​
What is your annual household income?	Less than $20,000; $20,000–$34,999; $35,000–$49,999; $50,000–$74,999; $75,000 or more
What is the highest level of education you have completed?	Less than high school; High school graduate or equivalent (e.g., GED); Some college or vocational training; Bachelor's degree; Graduate or professional degree
How many children do you have?	Open-ended
What are the ages of your children?	Open-ended
Have you ever declined or delayed any recommended childhood vaccines for your child/children?	Yes/No
If yes, which vaccine(s) did you decline or delay?	Open-ended
What are the main reasons you chose to decline or delay vaccination for your child/children?	Open-ended
How much do you agree with the following statement: "Vaccines are safe for my child/children."	Strongly agree, Agree, Neither agree nor disagree, Disagree, Strongly disagree
How much do you agree with the following statement: "Vaccines are effective in preventing serious diseases."	Strongly agree, Agree, Neither agree nor disagree, Disagree, Strongly disagree
Where do you typically get information about vaccines?	Healthcare provider, Family or friends, Social media, Internet searches, Religious leaders, Other
How much do you trust the information you receive about vaccines from your healthcare provider?	Completely trust, Mostly trust, Somewhat trust, Slightly trust, Do not trust at all
What would make you more likely to accept recommended vaccines for your child/children?	Open-ended
Is there anything else you would like to share about your thoughts or experiences related to childhood vaccines?	Open-ended
